# Analyzing the Impact of Objects in an Image on Location Estimation Accuracy in Visual Localization

**DOI:** 10.3390/s24030816

**Published:** 2024-01-26

**Authors:** Sungho Moon, Myungho Lee

**Affiliations:** 1 Department of Information Convergence Engineering, Pusan National University, Busan 46241, Republic of Korea; moonsungho22@pusan.ac.kr; 2 School of Computer Science and Engineering, Pusan National University, Busan 46241, Republic of Korea

**Keywords:** visual localization, augmented reality, semantic segmentation, synthetic dataset

## Abstract

Visual localization refers to the process of determining an observer’s pose by analyzing the spatial relationships between a query image and a pre-existing set of images. In this procedure, matched visual features between images are identified and utilized for pose estimation; consequently, the accuracy of the estimation heavily relies on the precision of feature matching. Incorrect feature matchings, such as those between different objects and/or different points within an object in an image, should thus be avoided. In this paper, our initial evaluation focused on gauging the reliability of each object class within image datasets concerning pose estimation accuracy. This assessment revealed the building class to be reliable, while humans exhibited unreliability across diverse locations. The subsequent study delved deeper into the degradation of pose estimation accuracy by artificially increasing the proportion of the unreliable object—humans. The findings revealed a noteworthy decline started when the average proportion of the humans in the images exceeded 20%. We discuss the results and implications for dataset construction for visual localization.

## 1. Introduction

In augmented reality (AR), achieving robust registration of virtual objects in a user’s 3D space is a crucial feature, attainable through accurate pose estimation of the user’s head or the AR device’s camera [1,2]. Modern AR devices default to exploiting a technique called simultaneous localization and mapping (SLAM), coupled with various sensor fusion algorithms [3,4]. As its name implies, SLAM concurrently builds a map of the previously unseen environment while continuously estimating the device’s pose within the map. Lidar and cameras commonly serve as sensors for SLAM, with further categorization as visual SLAM when cameras are predominantly utilized, and in the case of a single camera, it is referred to as monocular SLAM [5]. Smartphone-based AR frameworks, such as ARCore and ARKit, predominantly utilize variants of monocular SLAM.

While visual SLAM provides sufficient tracking performance for room-sized indoor AR cases, it often falls short in large outdoor spaces. Apart from the unpredictable lighting conditions outdoors, which pose challenges for most color imaging sensors, the size of the space itself can be problematic. For instance, to achieve localization, a robust and reliable map must be constructed. For this purpose, visual features in the space need to be observed multiple times from various angles for a so-called bundle adjustment procedure [6]. However, users may only cover sub-areas of the space, and in the worst-case scenario, they might follow a linear path, resulting in high uncertainties in the poses of the visual features. The consideration of alternative solutions, such as long-range lidar or GPS receivers for outdoor AR scenarios, may arise. However, long-range lidars, while effective, are often too bulky for integration with AR headsets or smartphones, and GPS suffers from significant pose estimation errors.

The fundamental concept behind visual localization (VL) contrasts with the dynamic map-building approach, suggesting the notion of constructing the map in advance [7,8]. Instead of generating a map on the fly, VL relies on a pre-built, extensive image dataset. With this pre-built dataset, VL creates a 3D structural map from the images or assigns relative poses to each image for later use. When a query image is fed, a VL algorithm extracts visual features from the image and identifies matches with those in the pre-built dataset, leading to a Perspective-n-Point problem for pose estimation [9]. Consequently, VL can be effectively applied in both large indoor and outdoor settings, given sufficient images are captured from the environments. Additionally, it is compatible with devices equipped with only a single camera, which includes the majority of smartphones.

In this paper, our focus delves into assessing the sufficiency of the image dataset for VL. Instead of merely considering the number of images in the dataset, we concentrate on the influence of the objects included in the dataset. Non-uniform surfaces, dynamically changing shapes, or textures are types of properties an object can have, which may result in generating different sets of visual features from an identical object under varying lighting conditions and viewing angles, disrupting feature matching. Furthermore, moving objects appearing in multiple images could further degrade pose estimation. Thus, the reliability of each object, in terms of pose estimation, could differ.

In the initial experiment, we examined the frequency of object appearances in VL datasets and assessed the reliability of each object class. To achieve this, we used an outdoor dataset consisting of six distinct locations for VL and conducted semantic segmentation on the images in the dataset. We identified the most frequently appearing object classes and further explored their effects on pose estimation performance. This was achieved by masking each class during the feature extraction phase of VL. Across all the locations, the human class consistently demonstrated negative impacts on pose estimation accuracy. We also investigated to what extent the inclusion of humans should be permissible in dataset creation, considering the impracticality of completely removing humans from spaces. For this purpose, we created synthetic datasets in which we controlled the average proportion of pixels occupied by humans in the images. The experiment confirmed that pose estimation accuracy decreased as the average proportion of humans in the dataset increased. A significant drop in performance was observed when this proportion exceeded 20%.

The rest of this paper is organized as follows: Section 2 presents previous work on visual localization, encompassing visual features and datasets. Section 3 and Section 4 detail the two experiments conducted. These include assessing object reliability and determining the allowable proportion of humans in VL datasets, with a discussion on implications for feature extraction and dataset creation in VL. Finally, we conclude the paper and discuss future work in Section 5.

## 2. Related Work

### 2.1. Visual Localization Method

Visual localization can be categorized into three methods: the structure-based method, the absolute pose estimation method (APE), and the relative pose estimation method (RPE).

Structure-based methods [10,11,12,13] estimate camera pose through 2D-3D matching between 3D points in a point cloud obtained using structure from motion [14,15] and local features in an image. This matching is mainly performed by calculating the geometric relationship and solving the Perspective-n-Point [16] problem. Recently, research has been conducted to perform 2D-3D matching through Deep Neural Networks [17,18,19,20]. The structure-based method has the advantage of high accuracy in pose estimation because it uses geometric relationships through dense point clouds. However, it has the disadvantage of requiring a significant amount of computation time as it needs to perform 2D-3D matching with a large number of 3D points. In addition, it is not possible to estimate the locations of points that were not captured during the point cloud creation process. For this reason, it is not suitable for AR, which requires real-time pose estimation with a mobile device over a large space.

APE represents a method where the traditional pose estimation process is entirely replaced by AI-driven techniques [21,22,23,24,25,26]. APE mainly uses Convolutional Neural Networks (CNNs), such as GoogLeNet [21,22] and ResNet [24], utilizing an end-to-end method that inputs an image and outputs its pose. APE has the advantage of a simple process and low computational effort because it uses only one AI model to estimate the pose of the image. However, when the location to be estimated changes, the AI model requires retraining, and since its pose estimation accuracy is lower than that of other techniques [27], it is not yet suitable for AR applications.

RPE performs pose estimation by analyzing the relative poses between images [28,29,30,31]. In RPE, a set of images comprises reference images, which contain pose information about the space where the pose estimation is conducted, and query images, whose poses are to be estimated. Additionally, RPE utilizes two types of features for matching: global features, representing the overall characteristics of the images, and local features, highlighting specific, important points within the images. Global features are used to select reference images similar to the query image, while local features are used to estimate the relative pose of the query image to the reference images, employing either geometric relations [28,29] or AI [32,33]. Global-feature-based image similarity comparison utilizes cosine similarity, which requires less computation and enables fast estimation, even with a large number of images. Furthermore, local-feature-based relative pose estimation uses a fixed, relatively small number of images selected based on global feature similarity. Therefore, RPE has the advantage of estimating the pose in real time with reduced computation, making it suitable for use in large areas. Additionally, since poses are estimated through the relative poses between images, it is possible to estimate the pose, to some extent, even for viewpoints not captured during the data collection process.

While RPE, compared to other VL methods, appears suitable for outdoor AR, it still faces many challenges. RPE’s pose estimation relies on matching local features between the query image and reference images. Accurate pose estimation can be difficult if the local features at the same location change. Common causes of such changes include variations in lighting [34], weather, or seasons [35] as well as the inherent dynamics of objects, like the movement of tree leaves. Additionally, the appearance or disappearance of moving objects, such as pedestrians or vehicles, can obscure important local features of the background or introduce new features, leading to incorrect pose estimation [36]. Therefore, this paper focuses on analyzing each object’s impact on visual localization, with the aim of improving estimation performance in the context of RPE-based VL. For convenience, we will refer to VL utilizing the RPE method simply as VL in the following sections of the paper.

### 2.2. Local Features

The accuracy of VL in pose estimation varies depending on the types of features used for image matching; therefore, extracting consistent features across diverse scenarios is essential for precise position estimation. Traditionally, procedural algorithms like SIFT [37] or Harris corner [38] were employed for feature-point extraction. However, a challenge emerged as the extracted feature points were prone to change due to factors such as variations in illumination from day to night or weather or seasonal changes. To address this issue, recent efforts have focused on data-driven approaches using deep learning for feature-point extraction [39,40,41,42]. While initial efforts aimed to replicate procedural features, recent research has shifted towards using various loss functions such as triplet loss [43] and Average Precision (AP) loss [40,44] to enhance feature extraction. Furthermore, recent research suggests extracting features based on their reliability and focusing on repeated patterns or objects in the image rather than indiscriminately extracting features from all areas.

One such AI-based feature extractor is the Repeatable and Reliable Detector and Descriptor, known as the R2D2 [40]. The main idea of the R2D2 is to reduce the reliability of repeated patterns or featureless areas in an image, thus extracting features that are valid for image matching. It exploits unsupervised learning so that no features are extracted from those unreliable areas. The R2D2 learns a reliability map that represents the reliability of an image and a descriptor together, dividing the image into multiple patches and matching each patch with a patch of another image to calculate the Average Precision (AP) for each patch. In this process, the threshold value k is set for the AP loss to reduce the reliability of patch areas with an AP lower than k to 0 while increasing the reliability of those with an AP higher than k to 1. The authors demonstrated that the R2D2 outperformed existing feature extractors by reducing the likelihood of extracting features from unreliable areas, like the sky and water, while enhancing feature extraction from reliable areas, such as buildings.

While the R2D2 uses image patches without intending to distinguish individual objects, the authors of BiasAttNet focused on features from individual objects [32]. They proposed a pipeline consisting of two main modules: the Bias Net and the Attention Net. This pipeline performed semantic segmentation to extract the region of each object using PSPNet [45], then trained the weights of each object through the Bias Net, which consists of a CNN layer. Subsequently, the Attention Net, consisting of a Residual Network and Convolution Block Attention Module [46], extracts local features. The authors demonstrated that the proposed pipeline improved pose estimation accuracy. However, their results were confined to the inclusion of the building class, without specific reasons provided, while other object classes were left unexplored.

Thus, in this study, we aim to extend the reliability assessment to different object classes commonly appearing in VL datasets. In Experiment 1, we examine how the pose estimation performance varies by excluding each class of objects in the feature extraction phase.

### 2.3. Localization Dataset

VL has been significantly enriched by the creation of various benchmarking datasets. These datasets are designed to encompass a wide range of challenges in VL, such as spatial scale, illumination variations, weather and seasonal changes, as well as dynamic and moving objects. A typical VL dataset is composed of query images and reference images, with baseline pose estimates or 3D models for validating VL models and methods. Depending on the target applications, e.g., AR or autonomous driving, various indoor and/or outdoor spaces have been captured for VL datasets.

Outdoor datasets typically encompass a broad range of environmental conditions. The RobotCar Seasons dataset [35], comprising images in Oxford, UK, spans an entire year and captures diverse seasonal and weather conditions, rendering it invaluable for scenarios like autonomous driving, where accurate localization against a potentially outdated reference scenes is crucial. In contrast, the Aachen Day-Night dataset [34], featuring the city of Aachen, Germany, focuses on the day–night dichotomy. It provides a robust platform for testing nighttime image localization against daytime 3D models, crucial for understanding outdoor localization amidst varying weather, seasonal, and day–night cycles, especially with pedestrian presence. Notably, images in this dataset were collected using mobile devices, making it especially useful for AR applications. The Cambridge Landmarks dataset [21] adds another dimension to outdoor urban localization. Covering six distinct areas around Cambridge University, this comprehensive dataset includes original videos, extracted image frames labeled with their 6-DOF camera poses, and visual scene reconstructions essential for large-scale and detailed visual localization in urban settings.

While various environmental conditions are included in outdoor datasets, the Gangnam Station and Hyundai Department Store dataset [36] specifically addresses challenges posed by crowded indoor environments. Collected in high-density urban settings, such as department stores and subway stations, it highlights the impact of human presence on VL performance. The authors’ initial investigation into how crowd density affects pose estimation revealed a decrease in performance for crowded images, defined as those where 20% of pixels represent people, in comparison to less crowded images. They concluded that human presence interferes with pose estimation. Investigating the effect of crowdedness on VL performance is essential for dynamic, densely populated indoor spaces, where human movement and interaction significantly influence pose estimation accuracy. However, the decrease in VL performance reported might also stem from occlusions or interference from other objects, underscoring the need for further research. Experiment 2 mainly addresses this issue.

## 3. Experiment 1

In this experiment, we assessed the reliability of each object class for visual localization.

### 3.1. Dataset

**Figure 1:**
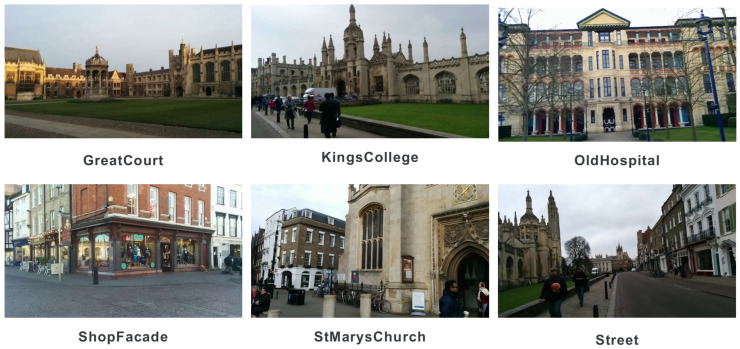
Sample images from the six locations included in the Cambridge Landmarks dataset.

Given the variety of locations and objects, we selected the Cambridge Landmarks dataset for this experiment. This dataset includes a total of 8454 reference images and 4864 query images, distributed across six locations: Great Court, King’s College, Old Hospital, Shop Facade, St. Mary’s Church, and Street. Table 1 illustrates the image count in the dataset, and Figure 1 displays example images from each location.

### 3.2. Selected Object Classes

**Figure 2:**
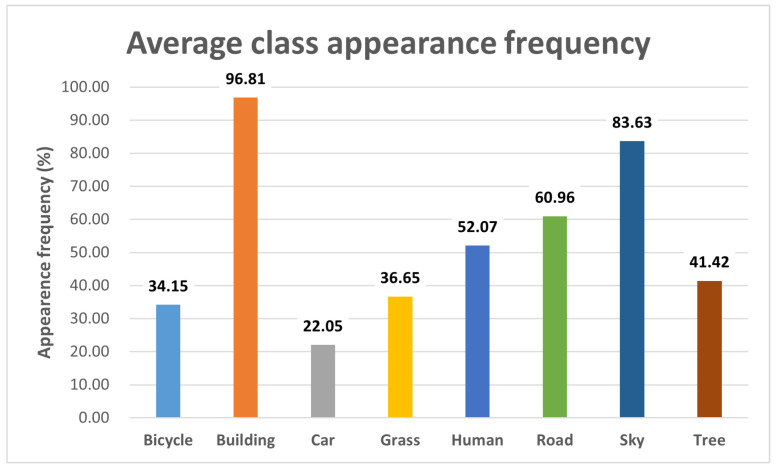
The average appearance frequency distribution for each object class.

We first analyzed the frequency of each object’s appearance in the dataset to select specific object classes for further investigation. For this task, we employed SEEM [47], a multimodal semantic segmentation AI, which enabled us to accurately classify objects. During this process, we aggregated similar classes, such as motorbikes and bicycles, for a more streamlined analysis. Ultimately, we chose to focus on object classes that appeared in more than 20% of the images. This led us to identify eight primary classes for our detailed assessment: bicycle, building, car, grass, human, road, sky, and tree. The frequency distribution of these selected object classes is presented in Figure 2.

### 3.3. Class-Masked Visual Localization

We conducted a pose estimation performance analysis using the selected object classes by excluding feature points from each object class during pose estimation. For this analysis, we adopted the RPE method and implemented VL using Kapture, an open-source framework developed by Naver Labs [29]. We employed AP-GeM [48] for global feature extraction to select reference images from the dataset, with a configuration set to choose five reference images per query image. Subsequently, local features from the query and reference images were extracted and matched using the R2D2 [40].

**Figure 3:**
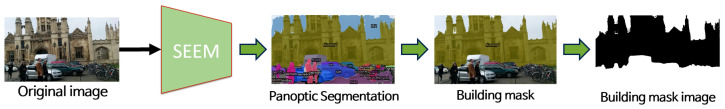
The process of creating class-specific mask images, illustrated with the building class as an example.

For class-specific masking, we utilized SEEM. Figure 3 demonstrates the process of creating a mask image for the building class using SEEM as an example. The initial step involved performing panoptic semantic segmentation on the dataset images, which classified all pixels into 133 distinct classes. Subsequently, we isolated pixels associated with the eight selected classes to create specific masks for each class. During the extraction of local features from these images, we utilized these class masks to prevent the extraction of local feature points from the masked areas. To assess the impact of each class on pose estimation accuracy, we compared results with and without the exclusion of each object class.

### 3.4. Results

In this study, we evaluated pose estimation performance using a metric commonly applied in VL research: the percentage of query images whose pose estimation error falls within a specific threshold relative to the total number of query images [34]. We set our thresholds at 0.25 m for positional error and 2.0 degrees for rotational error. As our primary aim was to analyze performance changes, we compared the performance metrics of VL with and without class-specific masking for each class. This comparison involved calculating the difference in the percentage of query images within the threshold out of the total query images for each class across six distinct locations.

Table 2 displays the changes in localization performance for each object class. The results indicated a decrease in pose estimation performance for the building class, while there was an increase for the bicycle, car, grass, human, road, sky, and tree classes. Notably, the human and car classes were the only categories showing consistent performance improvement across all locations.

### 3.5. Discussion

In this experiment, we focused on analyzing changes in VL performance by masking eight prominent object classes during local feature extraction, aiming to assess the reliability of each object class in VL. The results showed that the building class was the most reliable among the tested classes. It demonstrated a significant decrease in localization performance when masked, a trend consistent across all six locations. On the other hand, the car and human classes had a negative impact on localization performance in all locations, rendering them unreliable for VL. While these findings were somewhat anticipated, they offer objective and empirical support for the inclusion or exclusion of specific object classes in VL datasets, as seen in previous studies.

We also noted considerable variations in the impact—the performance changes—of different object classes. We speculate that this might be related to the proportion of image pixels occupied by each class. Our analysis indicated that the building class accounted for an average of 51.63% of image pixels, compared to only 0.69% and 1.15% for the car and human classes, respectively. This disparity raises a question about the acceptable pixel ratio for less reliable objects when creating VL datasets, especially in locations where evacuating the area for dataset collection is impractical.

## 4. Experiment 2

AR typically involves an individual wearing AR devices and viewing virtual content or objects superimposed onto a real-world background. Unlike autonomous driving, the spaces where VL occurs in AR are areas where humans are present. Consequently, images captured in these spaces are highly likely to include pedestrians. In this experiment, we aimed to investigate two key aspects: (1) whether increasing the proportion of the unreliable object class, specifically humans, in a VL dataset leads to a decrease in localization performance and (2) to what extent the inclusion of humans should be allowed in a VL dataset. To this end, we synthetically added humans to the reference images in a VL dataset to increase their proportion while keeping the query images unchanged.

To quantify the proportion of humans in a dataset, we defined the Human Pixel Ratio (HPR), adopting the concept of “crowdedness” from the previous work by Lee et al. [36]. The HPR measures the proportion of pixels in an image that represent humans. For this experiment, we utilized Yolo v7 [49] for human segmentation instead of SEEM, owing to its superior segmentation quality with the dataset used in our study. For the pose estimation, we utilized the same VL implementation as described in Section 3.3, which was carried out using Kapture with AP-GeM as the global feature extractor and the R2D2 as the local feature extractor.

### 4.1. Base Dataset

In this experiment, we opted for the Aachen Day-Night dataset [34] as our base dataset to create Human-Synthesized datasets with varying HPRs. This decision was driven by the need to minimize repeated use of the same human figures in the synthesis. Such repetition was a challenge with the Cambridge Landmark dataset, which had a limited number of images containing humans in certain locations.

The Aachen Day-Night dataset comprises 6848 reference images, 824 daytime query images, and 191 nighttime query images. Our preliminary analysis showed that 3881 of the reference images featured humans, averaging an HPR of 2.25%. We then examined the influence of human presence on pose estimation within this dataset. This involved counting the total and human-inclusive matched reference images as well as the local feature points matched between query and reference images, specifically those within human-occupied areas. It should be noted that we aggregated the data obtained from all Aachen Day-Night query images by summing them respectively. Additionally, the number of reference images set for pose estimation was limited to five. Table 3 summarizes the results with the base dataset. It shows that while humans appeared in 14.48% of the matched images, the actual use of local feature points located in human areas for matching was a mere 0.361%.

### 4.2. Derived Datasets

#### 4.2.1. Human Color-Changed Dataset

**Figure 4:**
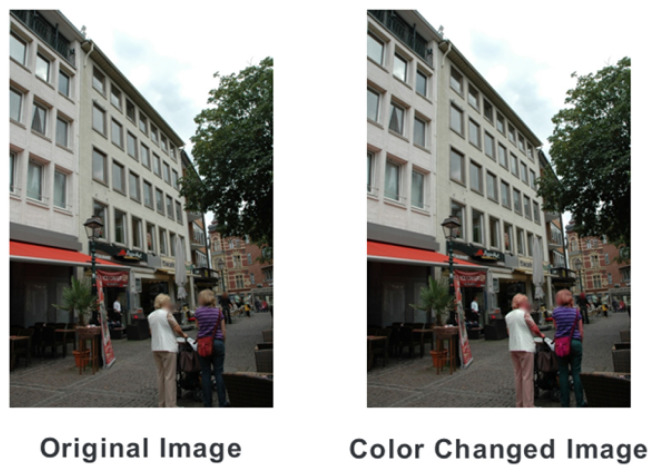
Example of the human color-changed dataset.

Due to the varying lighting conditions in each image, the color of the synthesized humans may not always match the background. If such color discrepancies between the synthesized humans and the background affect pose estimation accuracy, conducting a proper analysis with synthesized images becomes challenging. To address this, we created a color-modified dataset by altering the color of the human regions in the existing images. This process involved extracting the human figures from the images, applying color transformations, and then re-synthesizing them into the same positions, intentionally creating color inconsistencies between the humans and their surroundings. For the color variations in the human region, we utilized TensorFlow’s color jitter function. Since we synthesized the same humans in identical locations, the HPR remained unchanged. Figure 4 shows examples from this modified dataset.

#### 4.2.2. Human-Synthesized Dataset

**Figure 5:**
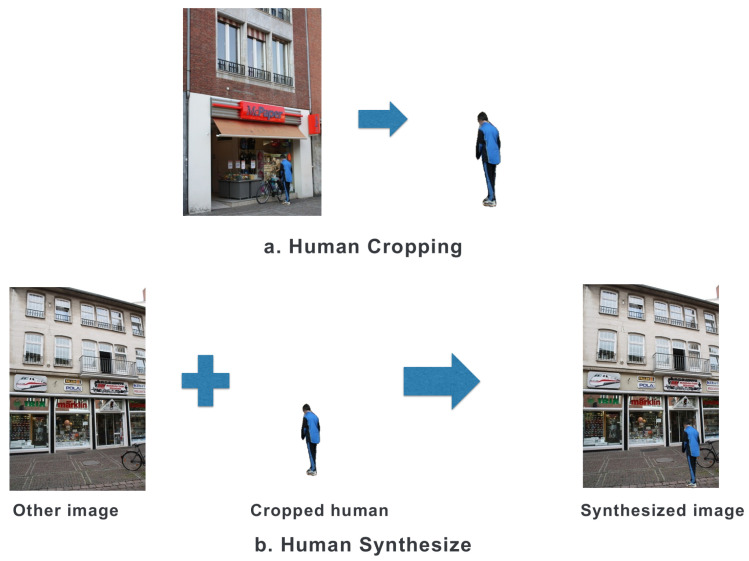
Process of creating the Human-Synthesized dataset.

To investigate the impact of HPR on VL performance, we generated the Human-Synthesized dataset by adding additional human figures to the reference images in the original dataset. To ensure a natural integration of the synthesized humans with the background, we selected human-containing reference images from the original dataset, focusing on those with a pixel ratio above 0.5% to avoid incorporating only partial human figures. Considering a typical AR application scenario, where images are often captured at eye level, we positioned the synthesized humans at the same ground level as the capturing human. Consequently, we placed these humans towards the bottom of the images. Figure 5 shows the process of creating the Human-Synthesized dataset.

**Figure 6:**
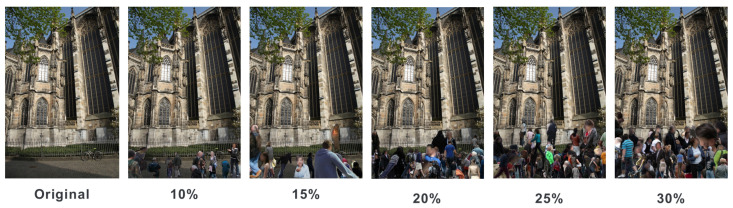
Sample images from the Human-Synthesized datasets at various HPRs.

Using this approach, we continuously synthesized extracted human images into the original images until the desired HPR was achieved. We allowed for a tolerance of ±1% deviation in each image and created datasets with average HPRs of 10%, 15%, 20%, 25%, and 30%. Figure 6 displays examples from these resulting datasets.

### 4.3. Evaluation Metrics

Similar to Experiment 1, we assessed changes in localization performance by comparing the base dataset with each derived dataset. Performance was gauged using the percentage of query images with pose estimation errors within specific thresholds relative to the total number of query images. The thresholds were set at 0.25 m for positional error and 2.0 degrees for rotational error.

Additionally, we introduced the Feature Ratio to evaluate the impact of synthetically added humans on VL. The key to pose estimation lies in calculating the relative pose through feature matching. The inclusion of human figures in the images may not always influence pose estimation as feature points in human areas might be excluded from matching due to the characteristics of descriptors or RANSAC algorithms that filter out incorrect matches [29]. In such cases, the human figures only contribute to occlusion. Consequently, we defined the Feature Ratio as the percentage of feature points from human areas in the reference images used for matching among all feature matching pairs. We then examined how the Feature Ratio varied with changes in HPR.

### 4.4. Results

#### 4.4.1. Color-Changed Dataset

**Figure 7:**
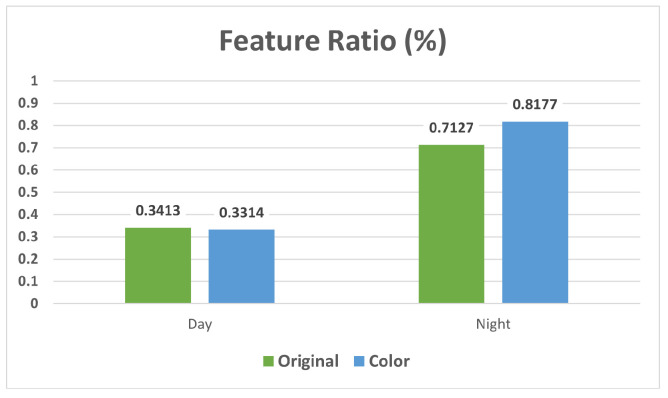
Changes in Feature Ratio associated with color changes.

In the Color-Changed dataset, we observed performance changes of 0.6% for day query images and 0.5% for night query images. As for the Feature Ratio, it altered by 0.1% in night query images and only 0.01% in day query images, as shown in Figure 7. These results suggest that color discrepancies between synthesized humans and their surroundings in the images have a negligible effect on feature matching. Furthermore, the higher Feature Ratio during night queries indicates a greater impact of synthesis compared to day queries.

#### 4.4.2. Human-Synthesized Datasets

**Figure 8:**
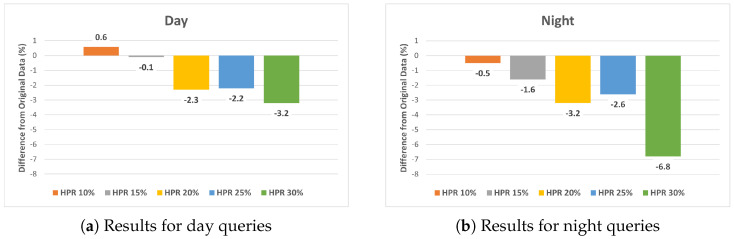
Comparative analysis of localization accuracy differences between the original dataset and the Human−Synthesized datasets for (**a**) day and (**b**) night queries.

Our findings reveal a trend of diminishing localization accuracy with an increasing HPR in both day and night query images within the Human-Synthesized datasets. Figure 8 summarizes these results. It is important to note that these scores were obtained by subtracting the performance of the base dataset from each synthesized dataset. In both day and night query images, a significant decrease in performance was observed at HPRs of 20% or higher.

**Figure 9:**
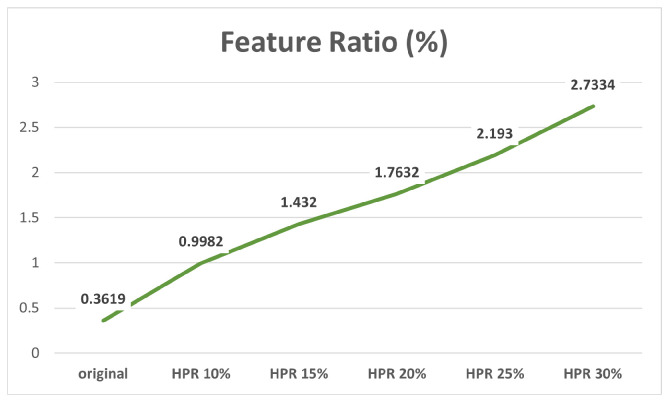
Changes in Feature Ratio with increasing HPR.

Regarding the Feature Ratio, our data indicated a consistent increase in Feature Ratio as HPR increased (see Figure 9). In other words, the more humans present in a VL dataset, the higher the likelihood of using feature points from human areas in pose estimation, potentially leading to errors in estimation accuracy.

Despite the observed increase in the Feature Ratio, there remains a question as to whether the observed decrease in localization accuracy is solely a result of occlusion by humans or due to mismatches caused by unreliable feature points from human areas. To explore this issue, we undertook a comparative performance analysis by applying feature masking to the human regions in the images. This analysis was particularly focused on the dataset with a 30% HPR, where the impact of human-induced occlusion is most pronounced.

**Figure 10:**
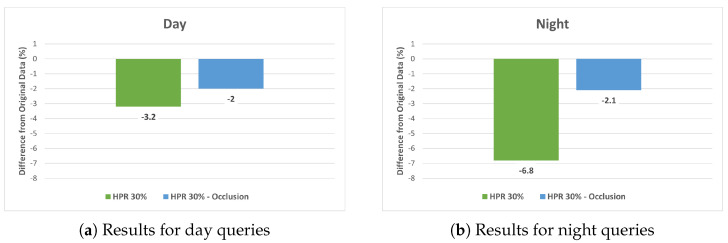
Localization accuracy comparison at 30% HPR: Human−Synthesized vs. occlusion for (**a**) day and (**b**) night queries.

Figure 10 depicts the differences in localization accuracy between the HPR 30% Human-Synthesized dataset and the equivalent dataset with human feature masking (HPR 30% occlusion). The results indicated a decline in performance attributable to occlusion, yet this decline was less pronounced than in the Human-Synthesized dataset. The disparity was especially evident in the night query images.

### 4.5. Discussion

In this experiment, we discovered that an increase in HPR leads to a decrease in localization accuracy, with a more pronounced decline observed in night query images. This suggests that the presence of humans significantly affects VL performance, particularly under low-light conditions.

The reduction in localization accuracy was not solely due to occlusion by humans obscuring reliable feature points. Our investigation, involving feature masking in human regions in datasets with 30% HPR, indicated that performance changes also stemmed from incorrect feature matching caused by feature points from human areas. This finding is crucial as it differentiates the impact of human presence from simple occlusion.

However, completely excluding humans while constructing a VL dataset is practically challenging. Therefore, we required a threshold for performance changes to establish an appropriate HPR. Based on the localization performance changes under various local feature extractors reported in the work of Lee et al. [36], we calculated an average difference of 2.1% and set it as an indicator of significant performance change. With this threshold, our results suggest that images for VL should contain less than 20% human occupancy to maintain accuracy. Filtering out images with more than 20% of pixels occupied by unreliable objects, such as humans, can enhance the reliability of VL datasets. This approach is particularly relevant for AR scenarios, where objects frequently appear and disappear.

Additionally, our findings can be applied to dataset creation through synthesis, especially in scenarios that are difficult to capture directly, like crowded spaces or the sudden appearance of obstacles. Our experiment confirms that synthesis itself does not significantly impact localization performance, thereby establishing it as a viable method for creating datasets in challenging situations.

## 5. Conclusions and Future Work

In this study, we conducted two distinct experiments aimed at evaluating the reliability of various object classes for VL and examining the permissible proportions of unreliable objects in VL datasets. In the first experiment, we identified eight common object classes—bicycle, building, car, grass, human, road, sky, and tree—and assessed their impacts on VL performance. Our results revealed the building class as a reliable object class, whereas the car and human classes were identified as unreliable. Considering the typical AR scenario where VL datasets are often generated in human-populated areas, we further analyzed the influence of human presence at various inclusion ratios. We utilized the HPR to quantify the proportion of pixels occupied by humans in the dataset and created datasets with varying HPRs by synthetically adding human figures. The results indicated that humans adversely affect localization performance, with significant degradation observed starting from an HPR of 20%. Furthermore, we determined that the decrease in localization accuracy was primarily due to the introduction of unreliable feature points from human areas, although occlusion by humans also played a role.

While these findings are valuable for practitioners and researchers developing VL datasets, it is important to note some limitations. First, the object classes we investigated are specific to our chosen dataset, which covered six different locations. The range of common object classes and their reliability might vary depending on geographical and urban factors. Second, our study focused exclusively on outdoor datasets, while AR applications are often used in indoor environments, highlighting the need for an analysis specific to indoor settings.

In future work, we aim to address these limitations and further enhance our approach. Our plan includes developing a more sophisticated synthesis method that not only considers the locations within an image but also leverages an understanding of structures or land use, such as stairs, rooftops, or crosswalks, appearing in the image. Additionally, we intend to train the R2D2 to utilize class masks based on the assessed reliabilities of each object class, incorporating these masks into the R2D2’s reliability map.

## Figures and Tables

**Table 1:** The number of images per location.

	Reference	Query	Total
GreatCourt	1533	761	2294
KingsCollege	1220	345	1565
OldHospital	902	182	1084
ShopFacade	231	103	334
StMarysChurch	1488	530	2018
Street	3080	2943	6023

**Table 2:** Changes in localization performance by object class when excluded.

	Bicycle	Building	Car	Grass	Human	Road	Sky	Tree
GreatCourt	-	−39.16	-	0.47	0.47	0.05	0.29	0.31
KingsCollege	−0.06	−43.03	0.06	−0.23	0.53	0.00	0.12	−0.17
OldHospital	-	−49.12	-	−0.11	-	0.11	1.21	4.39
ShopFacade	0.39	−22.33	0.00	-	0.19	0.39	−0.78	0.39
StMarysChurch	−0.08	−33.74	-	-	0.00	−0.49	−0.53	−0.80
Street	0.17	−39.08	0.27	−0.04	0.60	0.68	0.13	0.22
Average	0.11	−37.74	0.11	0.02	0.36	0.12	0.07	0.72

**Table 3:** The number of matched reference images and feature points in pose estimation using the Aachen Day-Night dataset.

	Day	Night
Total Matched Images	3829	557
Matched Images with Human Presence	561	74
Total Matched Feature Points	249,314	14,592
Feature Points in Human Areas	851	104

## Data Availability

Data are contained within the article.
